# An assessment of the efficacy of searching in biomedical databases beyond MEDLINE in identifying studies for a systematic review on ward closures as an infection control intervention to control outbreaks

**DOI:** 10.1186/2046-4053-3-135

**Published:** 2014-11-11

**Authors:** Yoojin Kwon, Susan E Powelson, Holly Wong, William A Ghali, John M Conly

**Affiliations:** 1 Health Sciences Library, Libraries and Cultural Resources, University of Calgary, HSC 1450, Health Sciences Centre, 3330 Hospital Drive NW, Calgary, Alberta T2N 4N1, Canada; 2 The Ward of the 21st Century (W21C), W21C, GD01 TRW Building, 3280 Hospital Drive NW, Calgary, AB T2N 4Z6, Canada; 3 Departments of Medicine and Community Health Sciences, Institute for Public Health, Cumming School of Medicine, University of Calgary, 3280 Hospital Drive NW, Calgary, Alberta T2N 4Z6, Canada; 4 Institute for Public Health, University of Calgary, 3280 Hospital Drive NW, Calgary, Alberta T2N 4Z6, Canada; 5 Infection Prevention and Control, Calgary and Area, Alberta Health Services, 1403 29 St NW, Calgary, Alberta T2N 2T9, Canada; 6 Departments of Medicine and Microbiology, Immunology & Infectious Diseases, Institute for Public Health and Snyder Institute for Chronic Diseases, Cumming School of Medicine, W21C GD01 TRW Building 3280 Hospital Drive NW, Calgary, AB T2N 4Z6, Canada

**Keywords:** Bibliographic databases, MEDLINE, Embase, Information retrieval, Bibliometrics, Sensitivity, Specificity, Systematic review

## Abstract

**Background:**

The purpose of our study is to determine the value and efficacy of searching biomedical databases beyond MEDLINE for systematic reviews.

**Methods:**

We analyzed the results from a systematic review conducted by the authors and others on ward closure as an infection control practice. Ovid MEDLINE including In-Process & Other Non-Indexed Citations, Ovid Embase, CINAHL Plus, LILACS, and IndMED were systematically searched for articles of any study type discussing ward closure, as were bibliographies of selected articles and recent infection control conference abstracts. Search results were tracked, recorded, and analyzed using a relative recall method. The sensitivity of searching in each database was calculated.

**Results:**

Two thousand ninety-five unique citations were identified and screened for inclusion in the systematic review: 2,060 from database searching and 35 from hand searching and other sources. Ninety-seven citations were included in the final review. MEDLINE and Embase searches each retrieved 80 of the 97 articles included, only 4 articles from each database were unique. The CINAHL search retrieved 35 included articles, and 4 were unique. The IndMED and LILACS searches did not retrieve any included articles, although 75 of the included articles were indexed in LILACS. The true value of using regional databases, particularly LILACS, may lie with the ability to search in the language spoken in the region. Eight articles were found only through hand searching.

**Conclusions:**

Identifying studies for a systematic review where the research is observational is complex. The value each individual study contributes to the review cannot be accurately measured. Consequently, we could not determine the value of results found from searching beyond MEDLINE, Embase, and CINAHL with accuracy. However, hand searching for serendipitous retrieval remains an important aspect due to indexing and keyword challenges inherent in this literature.

## Background

Systematic reviews identify, appraise, and synthesize evidence that meets pre-specified criteria to answer a research question [1]. Comprehensive search strategies are critical; however, they can produce thousands of citations, with only a small number ultimately being included in the review [1-3]. This results in "inefficient use of valuable resources in terms of time involved in screening…a large number of records" [4]. Searching for information on the efficacy of an intervention that is mainly evaluated in observational studies is particularly challenging for indexers and searchers as methodologies are often poorly reported [5].

Prior research has indicated that although MEDLINE is capable of identifying the majority of relevant studies, searching this database alone for the purposes of conducting a comprehensive literature search is insufficient [6-18]. Interestingly, Golder and Loke found that MEDLINE was not necessary to retrieve all the relevant studies on drug adverse events [19]. However, the true value of the contribution of citations from other databases has often been questioned [20,21]. There are varying levels of coverage for different topics and subjects in different databases. From their analysis of the effectiveness of different databases in identifying studies for the WHO systematic review of maternal morbidity and mortality, Betrán et al*.* concluded that there is a "need for extending the search to other sources beyond well-known electronic databases" when conducting systematic reviews on this topic [6]. By assessing what is not retrieved when only one database is searched for literature on injury prevention and safety promotion, Lawrence reported that "no single database included all of the relevant articles on any topic and the database with the broadest coverage differed by topic" [12]. Golder et al. also worked on a topic without standardized terminology and concluded that "even sensitive search strategies with a broad range of synonyms may not identify all the references meeting the inclusion criteria that are available in a particular database" suggesting that searching a variety of sources is probably the most effective way to compensate for this [18]. Lorenzetti et al. explored the extent of contributions made by databases other than MEDLINE in rapid health technology assessments, and reached a similar conclusion in 2014 [17]. Likewise, Lemeshow et al. discovered that limiting the search for observational studies to one or two databases will retrieve only 60%–80% of relevant publications [8].

The purpose of our research was to audit the sources of studies used in an unpublished systematic review on the efficacy of ward closure as an infection outbreak control intervention measure, prepared by Wong H et al*.* (unpublished work) for Alberta Health Services. Our primary objective was to assess the effectiveness of searching additional scholarly biomedical databases beyond MEDLINE: what is the value of searching additional databases, and do the results from these databases impact the final conclusions of a systematic review of observational studies? Ultimately, we were unable to determine the impact of searching each database, but we did discover that hand searching is critical.

## Methods

### Data collection

We conducted a systematic review on the efficacy of ward closure as an infection control practice. In consultation with the primary investigators who are subject experts, databases were selected. The search strategy was developed by librarians. YK and SEP searched Ovid MEDLINE including In-Process & Other Non-Indexed Citations, Ovid Embase, CINAHL Plus, Cochrane Database of Systematic reviews (CDSR), LILACS, and IndMED for any study type discussing the implementation of ward closure in the case of an outbreak. CDSR was searched to identify studies that may not have been retrieved through the searches. The primary investigators were particularly concerned that the searches would retrieve international studies. LILACS, which indexes the scientific and technical literature of Latin America and the Caribbean, is recommended in the Cochrane Infectious Disease Group Guide to Search Strategy [22]. A Spanish speaking co-investigator provided lists of terms in Spanish and Portuguese and reviewed the abstracts in these languages. IndMED, which indexes selected peer-reviewed medical journals published in India, covering approximately 100 journals from 1985 onwards, was specifically requested by one of the primary investigators. YK and SEP also searched bibliographies of included articles, websites of the Centers for Disease Control and Prevention, the International Centre for Infectious Diseases, and the World Health Organization, and meeting abstracts from Community and Hospital Infection Control Association (2012 and 2013), International Consortium for Prevention & Infection Control (2012 and 2013), International Conference on Emerging Infectious Diseases (2010), Infectious Diseases Society of America (2012), European Society of Clinical Microbiology and Infectious Diseases (2012 and 2013), International Society for Infectious Diseases (2012), and Association of Medical Microbiology (2012). All citations were exported to RefWorks for bibliographic management. Studies were screened by two reviewers, including HW, first by title and abstract and then by full text using a pre-defined set of inclusion and exclusion criteria. To be included, a study had to meet the following inclusion criteria: (1) be set in tertiary acute care hospitals/facilities or long-term acute care hospitals; (2) indicate that complete or partial ward closure took place for at least 48 h for outbreak control. Publication types such as surveys, secondary data analysis, non-original reports, grey literature, editorials, letters, cost analyses, and reviews were excluded. No language or publication date range limit was applied.

### Data analysis

Using the same methodology as Betrán et al*.*, we recorded the source of each citation and tracked the number of references identified in each database as well as the number remaining after the removal of duplicates, all references included in the systematic review, and the databases that contained these references [6]. To determine whether the included studies were unique to the database from which they were retrieved or whether they were also present in other databases, we carried out title searches and recorded this information on a spreadsheet using Microsoft Excel 2010. We analyzed our search results using a relative recall method. As discussed by Lorenzetti et al*.*, relative recall is "the proportion of…relevant articles that any specific system, filter, or tool retrieves" [17]. For our study, the relative recall of each source was calculated by dividing the number of included citations retrieved from each database by the total number of citations included in the systematic review. In addition, all of the selected databases were searched for all titles in the set of included articles. The sensitivity of each search was calculated by dividing the number of included citations retrieved from each database by the total number of included articles indexed in each database.

## Results

A total of 2,095 unique citations were identified and screened for inclusion in the systematic review: 2,060 from database searching and 35 from hand searching and other sources. Out of these citations, 97 were included in the final review.

The yield of included studies from the selected databases ranged from 0% to 82.5% (Table 1). The MEDLINE search retrieved 80 (82.5%) of the articles included in the systematic review (Table 1). Out of these included articles, four were unique to MEDLINE (Table 1). However, 93 of the 97 articles included were indexed in MEDLINE (Table 1). The sensitivity of our search was 86% (Table 1). Our search in Embase was slightly more sensitive than MEDLINE; 91 of the included articles indexed in Embase, and searching the database also retrieved 82.5% of the articles included in the systematic review, which means that the sensitivity of our search in this database was 87.9% (Table 1). Seventy-six included articles overlapped between Embase and MEDLINE. Four were unique to Embase (Table 1). CINAHL retrieved 35 included articles and contributed four unique articles (Table 1). The sensitivity of the CINAHL search was 79.5% (Table 1). The LILACS search did not retrieve any of the included or unique articles but this database did index 75 of the 97 articles included in the systematic review (Table 1). Searching in IndMED did not retrieve any included articles or make any unique contributions (Table 1). Also, this database did not index any included article (Table 1). Even though all 97 included articles were indexed in at least one of the selected databases, eight were retrieved due to hand searching (Table 1).

**Table 1:** Number of articles identified by search strategies and included in systematic review

	**Number identified articles**	**Number of included articles retrieved by search strategy (*n *** **= 97)**	**Relative recall**	**Number of articles in systematic review indexed by each database (*n *** **= 97)**	**Sensitivity**	**Number identified after deduplication**	**Number of unique articles**
MEDLINE	832	80	82.5%	93	86%	777	4
Embase	1,024	80	82.5%	91	87.9%	361	4
CINAHL	294	35	36.1%	44	79.5%	99	4
LILACS	1,101	0	0%	75	0%	723	0
IndMED	165	0	0%	0	0%	88	0
Other	36	8	8.3%	-	-	36	8

## Discussion

We assessed the efficacy of searching beyond MEDLINE by reviewing the literature and analyzing our results from a systematic review of ward closure as an infection control intervention to control outbreaks, prepared by Wong H et al*.* (unpublished work). As this systematic review did not find any published controlled studies, the authors concluded that the implementation of ward closure for control of outbreaks should neither be actively encouraged nor discouraged, or formalized as a policy.

While Betrán et al*.* concluded that it is important to search regional databases to locate studies from journals not indexed in MEDLINE, Egger et al*.* argued that doing so "raises the worrying possibility that rather than preventing bias through extensive literature searches, bias could be introduced by including trials of low methodological quality" [6,20]. Egger et al*.* claimed, "trials that are difficult to locate tend to be of lower methodological quality than trials that are easily accessible and published in English," defining them as trials published in languages other than English, and in journals not indexed in MEDLINE [20].

Betrán et al*.* reported that their searches in MEDLINE, Embase, CINAHL, and LILACS were sensitive by 61.6%, 43.9%, 10.2%, and 5.3% respectively [6]. Our searches in MEDLINE, Embase, CINAHL, and LILACS, on the other hand, were sensitive by 86%, 87.9%, 79.5%, and 0% respectively (Table 1). In contrast to Betrán et al*.*’s recommendation about searching regional databases, the results of our data analysis in this specific subject area revealed that searching in databases other than MEDLINE, Embase, and CINAHL did not make such significant contributions; at only four unique citations each, Embase and CINAHL might also be considered to have contributed marginally. Our discovery of articles indexed in the databases but not retrieved by our search strategies supports Golder et al.’s findings that even the most sensitive search strategies will not retrieve all the relevant results when working with a subject lacking standardized terms and the "failure of search strategies…to identify all the relevant references available on each database" [18,19].

The LILACS results were particularly interesting. While LILACS indexed 75 articles included in the systematic review, none of our search strategies retrieved them. We initially searched this database using the same strategies as the other databases; however, as this did not yield any relevant articles, we modified our search using a few keywords provided by a Spanish speaker on the review team. The unique and potentially relevant articles located did not meet the inclusion criteria so were excluded from the systematic review. The true value of searching in LILACS may lie with the ability to carry out a search in Spanish or Portuguese. Clark and Castro do not explicitly address searching in English, Spanish, or Portuguese when claiming that LILACS could have added further information to 70% of the systematic reviews they surveyed, arguing that the "database should be used as a routine source of studies in the preparation of SR" [23]. According to Egger et al*.*, "systematic reviews that are based on a search of English language literature that is accessible in the major bibliographic databases will often produce results that are close to those obtained from reviews based on more comprehensive searches that are free of language restrictions" [20]. Future studies could investigate how to improve the specificity of a LILACS search and compare searching LILACS in English, Spanish, and Portuguese to confirm or refute Egger et al*.*’s claim.

One of the most noteworthy discoveries made during our data analysis was that database searches alone would have missed 8.3% of relevant citations. Although they were all indexed in one of the selected databases, we were only able to retrieve these citations through hand searching and reference checking. In general, this was because the focus of these articles was mostly on describing the overall experience of an infection outbreak and ward closure was not mentioned in the title, abstract, or keywords list. This suggests that time spent on "serendipitous means of identifying relevant information," rather than expanding the range of databases, was an efficient way to compensate for the citations that search in major databases failed to identify and was relevant to the topic area under review in this circumstance [18]. Specifically, strategies "such as…asking colleagues, pursuing references that look interesting, and simply being alert to serendipitous discovery," as Greenhalgh and Peacock argued, "may have a better yield per hour spent and are likely to identify important sources that would otherwise be missed" [24].

Although we demonstrate that the value in searching beyond the mainstream databases of MEDLINE, Embase, and CINAHL is marginal, our study does have limitations. In particular, as indicated previously, our review did not include any controlled study that could be used for a meta-analysis. Thus, quantifying the impact of each citation on the final result of the review and testing Betrán et al*.*’s or Egger et al*.*’s findings were not possible [6,20]. In addition, because our conclusions are specific to one systematic review of an intervention that was only described in observational studies, outcomes of interest may vary when searching for studies and reviews of other topics.

## Conclusions

Due to indexing and abstracting issues, using conventional protocol-driven search techniques in major bibliographic databases may lead to an inadvertent omission of significant articles in a systematic review of an intervention that requires extensive keyword searching. The results from our data analysis suggest that expanding the range of databases to search beyond MEDLINE, Embase, and CINAHL, however, may not be the most effective way to address this problem. Rather, redirecting effort into serendipitous discoveries may be a more efficient usage of the review team’s resources.

## Competing interests

The authors declare that they have no competing interests.

## Authors’ contributions

YK conducted literature search. YK and SP performed data analyses. All authors (YK, SP, HW, WAG, JMC) were involved in the manuscript development and its revision. All authors read and approved the final manuscript.

## References

[B1] Evidence-based health care and systematic reviews [ http://www.cochrane.org/about-us/evidence-based-health-care]

[B2] Dickersin K Scherer R Lefebvre C Systematic reviews: identifying relevant studies for systematic reviews BMJ 1994 309 6964 1286 1291 10.1136/bmj.309.6964.1286 7718048 PMC2541778

[B3] Moher D Liberati A Tetzlaff J Altman DG Preferred reporting items for systematic reviews and meta-analyses: the PRISMA statement Ann Intern Med 2009 151 4 264 269 10.7326/0003-4819-151-4-200908180-00135 19622511

[B4] Fraser C Murray A Burr J Identifying observational studies of surgical interventions in MEDLINE and EMBASE BMC Med Res Methodol 2006 6 1 41 10.1186/1471-2288-6-41 16919159 PMC1569861

[B5] Dalziel K Round A Stein K Garside R Castelnuovo E Payne L Do the findings of case series studies vary significantly according to methodological characteristics? Health Technol Assess 2005 9 2 iii iv 1–146 15588556 10.3310/hta9020

[B6] Betrán AP Say L Gülmezoglu AM Allen T Hampson L Effectiveness of different databases in identifying studies for systematic reviews: experience from the WHO systematic review of maternal morbidity and mortality BMC Med Res Methodol 2005 5 1 6 10.1186/1471-2288-5-6 15679886 PMC548692

[B7] Stevinson C Lawlor DA Searching multiple databases for systematic reviews: added value or diminishing returns? Complement Ther Med 2004 12 4 228 232 10.1016/j.ctim.2004.09.003 15649836

[B8] Lemeshow AR Blum RE Berlin JA Stoto MA Colditz GA Searching one or two databases was insufficient for meta-analysis of observational studies J Clin Epidemiol 2005 58 9 867 873 10.1016/j.jclinepi.2005.03.004 16085190

[B9] Wilkins T Gillies RA Davies K EMBASE versus MEDLINE for family medicine searches: can MEDLINE searches find the forest or a tree? Can Fam Physician 2005 51 6 848 849 16926954 PMC1479531

[B10] Ogilvie D Hamilton V Egan M Petticrew M Systematic reviews of health effects of social interventions: 1. Finding the evidence: how far should you go? J Epidemiol Community Health 2005 59 9 804 808 10.1136/jech.2005.034181 16100321 PMC1733146

[B11] Crumley ET Wiebe N Cramer K Klassen TP Hartling L Which resources should be used to identify RCT/CCTs for systematic reviews: a systematic review BMC Med Res Methodol 2005 5 24 10.1186/1471-2288-5-24 16092960 PMC1232852

[B12] Lawrence DW What is lost when searching only one literature database for articles relevant to injury prevention and safety promotion? Inj Prev 2008 14 6 401 404 10.1136/ip.2008.019430 19074247

[B13] Whiting P Westwood M Burke M Sterne J Glanville J Systematic reviews of test accuracy should search a range of databases to identify primary studies J Clin Epidemiol 2008 61 4 357 364 18313560 10.1016/j.jclinepi.2007.05.013

[B14] Slobogean GP Verma A Giustini D Slobogean BL Mulpuri K MEDLINE, EMBASE, and Cochrane index most primary studies but not abstracts included in orthopedic meta-analyses J Clin Epidemiol 2009 62 12 1261 1267 10.1016/j.jclinepi.2009.01.013 19364634

[B15] Fell DW Burnham JF Buchanan MJ Horchen HA Scherr JA Mapping the core journals of the physical therapy literature J Med Libr Assoc 2011 99 3 202 207 10.3163/1536-5050.99.3.007 21753912 PMC3133899

[B16] Beyer FR Wright K Can we prioritise which databases to search? A case study using a systematic review of frozen shoulder management Health Inf Libr J 2013 30 1 49 58 10.1111/hir.12009 23413793

[B17] Lorenzetti DL Topfer L-A Dennett L Clement F Value of databases other than Medline for rapid health technology assessments Int J Technol Assess Health Care 2014 30 2 173 178 10.1017/S0266462314000166 24774535

[B18] Golder S Mason A Spilsbury K Systematic searches for the effectiveness of respite care J Med Libr Assoc 2008 96 2 147 10.3163/1536-5050.96.2.147 18379671 PMC2268238

[B19] Golder S Loke YK The contribution of different information sources for adverse effects data Int J Technol Assess Health Care 2012 28 02 133 137 10.1017/S0266462312000128 22559754

[B20] Egger M Juni P Bartlett C Holenstein F Sterne J How important are comprehensive literature searches and the assessment of trial quality in systematic reviews? Empirical study Health Technol Assess 2003 7 1 1 76 12583822

[B21] Sampson M Should meta-analysts search Embase in addition to Medline? J Clin Epidemiol 2003 56 10 943 955 10.1016/S0895-4356(03)00110-0 14568625

[B22] Guide to the search strategy [ http://cidg.cochrane.org/sites/cidg.cochrane.org/files/uploads/search-strategy-guide_19MAR09_MODIFIED%208%20march%202013.pdf]

[B23] Clark OAC Castro AA Searching the Literatura Latino Americana e do Caribe em Ciencias da Saude (LILACS) database improves systematic reviews Int J Epidemiol 2002 31 1 112 114 10.1093/ije/31.1.112 11914305

[B24] Greenhalgh T Peacock R Effectiveness and efficiency of search methods in systematic reviews of complex evidence: audit of primary sources BMJ 2005 331 7524 1064 1065 10.1136/bmj.38636.593461.68 16230312 PMC1283190

